# The Rho GTPase regulator ARHGEF3 orchestrates hair placode budding by coordinating cell fate and P-cadherin patterning in mice

**DOI:** 10.1371/journal.pbio.3003572

**Published:** 2026-01-05

**Authors:** Krithika Kalyanakrishnan, Amy Beaudin, Alexandra Jetté, Sarah Ghezelbash, Diana Ioana Hotea, Jie Chen, Philippe Lefrançois, Mélanie Laurin

**Affiliations:** 1 Département de biologie moléculaire, biochimie médicale et pathologie, Faculté de médecine, Université Laval, Québec, Québec, Canada; 2 Oncology Research Program, CHU de Québec—Université Laval Research Center, Québec, Québec, Canada; 3 Centre de recherche sur le cancer de l’Université Laval (CRC), Québec, Québec, Canada; 4 Centre de recherche en organogénèse expérimentale (LOEX), Québec, Québec, Canada; 5 Cancer Axis, Lady Davis Institute for Medical Research, Montreal, Québec, Canada; 6 Division of Experimental Medicine, McGill University, Montreal, Québec, Canada; 7 Department of Cell & Developmental Biology, University of Illinois at Urbana-Champaign, Urbana, Illinois, United States of America; 8 Department of Biomedical and Translational Sciences, Carle Illinois College of Medicine, Urbana, Illinois, United States of America; 9 Division of Dermatology, Department of Medicine, McGill University, Montreal, Québec, Canada; California Institute of Technology, UNITED STATES OF AMERICA

## Abstract

During embryogenesis, cells self-organize into precise patterns that enable tissues and organs to acquire specialized functions. Despite its importance, the molecular choreography driving these collective cellular behaviors remains poorly understood, posing a major challenge in developmental biology and limiting progress in regenerative medicine. Here, we use the developing mouse hair follicle as a model mini-organ to investigate the early events of epithelial bud formation. We identify the Rho GTPase regulator ARHGEF3 as a critical upstream factor that restricts cell fate acquisition and establishes a radial gradient of P-cadherin across the placode during early hair follicle development. In *Arhgef3* knockout embryos, placodes are enlarged and exhibit elevated P-cadherin levels at cell-cell junctions, disrupting gradient formation without affecting E-cadherin distribution. This defect correlates with aberrant epithelial organization and increased incidence of straight hair follicle downgrowth. Our findings position ARHGEF3 as a novel regulator of cadherin patterning and placode polarization, and suggest broader roles in the morphogenesis of other epithelial appendages governed by similar developmental programs.

## Abstract 2

During embryogenesis, cells self-organize into patterns that support tissue-specific functions, but how these collective cellular behaviors are coordinated remains unclear. This study shows that the Rho GTPase regulator, ARHGEF3, regulates cadherin patterning and placode polarization in early hair follicle development, influencing epithelial organization and morphogenesis.

## Introduction

Hair follicle development in the mouse offers a powerful model to dissect how coordinated cell behaviors drive organogenesis. Their abundance, reproducible spatial and radial organization, and consistent alignment within the epidermal plane make hair follicles ideal for studying the molecular control of morphogenesis [1–4]. Hair follicle formation begins with the placode, an epithelial thickening that exemplifies a conserved strategy for generating new structures from an epithelium during development. Classical genetic studies have demonstrated that reciprocal signaling between the epidermis and dermis, involving key developmental pathways such as BMP, WNT, SHH, and FGF, sequentially regulates placode specification, polarization, budding, and differentiation [5,6]. More recently, tissue-intrinsic mechanical forces have also been implicated in shaping the architecture of the skin and its appendages [7–15]. Although live imaging has provided increasingly detailed views of the dynamic cellular choreography driving hair follicle morphogenesis [2,4], the molecular effectors that translate and execute upstream signaling and mechanical cues remain poorly defined.

**Fig 1:**
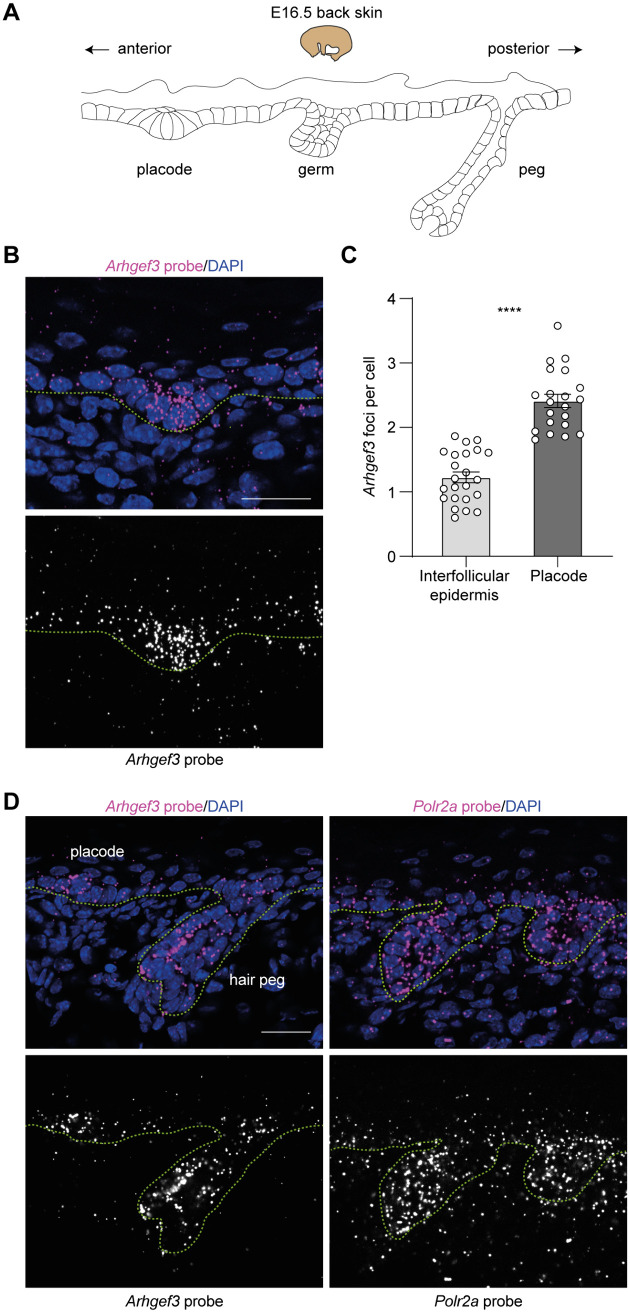
ARHGEF3 is expressed in the developing skin. **(A)** Schematic representation of hair follicle development. The hair placode, hair germ, and hair peg can all be observed in the back skin of E16.5 embryos due to the formation of staggered hair follicle waves during embryonic development. Asymmetric downgrowth of the hair follicle is characterized by an anterior tilt of the hair germ. **(B)** RNAscope in situ hybridization (ISH) targeting *Arhgef3* mRNA (magenta) in the E18.5 developing placode. **(C)** Graph displays the average number of *Arhgef3* foci per cell in the IFE or the placode at E18.5 as mean ± SEM. Statistical analyses were performed using a two-tailed Mann–Whitney test, *P* value < 0.0001, with placodes analyzed = 23 and IFE regions analyzed = 21 from one *Arhgef3*^+*/*+^ embryo and the experiment was performed once. **(D)** RNAscope in situ hybridization (ISH) targeting *Arhgef3* mRNA (magenta) in the E18.5 developing skin was performed. The sections at E18.5 show the maintenance of *Arhgef3* expression during different stages of hair follicle development and its expression in other skin compartments (*n* = 4 embryos of different embryonic stages and the experiment was independently performed 4 times). *Polr2a* mRNA is ubiquitously expressed in cells and was used as a positive control. Scale bars: 25 µm. DAPI was used to label cell nuclei. Dotted line is used to delineate the epidermis and hair follicles from the dermis. Underlying dataset for this figure is available at: https://doi.org/10.5281/zenodo.17400745.

The first distinct morphological sign of hair follicle development is the formation of the placode, which progresses into the hair germ and then the hair peg (**Fig 1A**). Both directional cell migration and cell compaction promote early placode formation [16]. Following placode cell elongation, contractile forces from the epidermis and dermis drive the placode cell basal membrane expansion while their apical surface remains constrained, leading to epithelial invagination [10]. As extracellular matrix remodeling occurs around the placode, mechanical pressure decreases, allowing placode cells to re-enter mitosis [10]. Further bud downgrowth is driven by oriented cell divisions, leading to the formation of hair germs and hair pegs [10,17]. Genetic manipulations, such as Myosin IIa deletion (*Myh9* knockout) or actomyosin inhibitor treatment in mouse skin explant, highlight the essential role of cytoskeletal regulation in these processes [2,10,13,14,16,18].

Cell fate in hair placodes emerges in a radial pattern, with concentric rings of distinct cell populations; namely an inner LHX2-expressing domain and an outer SOX9-expressing domain [2–4,19,20]. This patterning, driven by sequential WNT and SHH gradients, is essential for placode polarization, anterior tilting of the hair germ, and asymmetric progenitor repositioning [2,4]. These outcomes are achieved through coordinated counter-rotational cell rearrangements between neighboring cells that resemble convergent extension, a process that elongates tissue via cell intercalation and junctional shrinkage [2,21,22]. In fact, these rearrangements, and the resulting global alignment of hair follicles along the anterior-posterior axis of the mouse embryo, are governed by planar cell polarity (PCP), which refers to the coordinated polarization of a field of cells within the tissue plane [23,24]. During mouse skin development, PCP proteins such as CELSR1 become asymmetrically partitioned in basal epidermal and hair placode cells. This organization is essential for hair follicle orientation, as mutations in conserved PCP components, including *Frizzled-6*, *Celsr1* and *Vangl2,* impair PCP signaling and lead to hair follicle misorientation [24–29].

While PCP components may bias junctional contraction along a specific axis during placode neighbor exchange, an additional mechanism that promotes timely and robust cellular rearrangements involves the formation of opposing radial gradients of E-cadherin (Cadherin-1) and P-cadherin (Cadherin-3) across the placode [4]. These gradients arise through the upregulation of P-cadherin and concurrent downregulation of E-cadherin from the center of the placode outward [19,30,31]. This radial organization supports the anterior migration of inner LHX2^+^/P-cadherin-enriched cells, the posterior sliding of outer SOX9^+^ cells, and the overall polarization of the follicle [2,24]. Importantly, conditional knockout of E-cadherin (cKO) leads to compensatory upregulation of P-cadherin, abolishing the radial gradient and delaying placode polarization [4,32,33]. Conversely, E-cadherin overexpression impairs hair follicle formation by preventing epithelial invagination [30,34]. However, whether E- and P-cadherin-based adherens junctions recruit distinct effectors or activate unique cytoskeletal remodeling programs to facilitate neighbor exchange remains poorly understood.

Recently, we took advantage of our ability to transduce epidermal progenitors by injecting lentiviral particles into the amniotic cavities of mouse embryos using ultrasound guidance [35]. This method allowed us to conduct an RNAi-mediated screen aimed at identifying regulators of hair follicle morphogenesis among components of the Rho GTPase network, which are key cytoskeletal regulators [36–39]. Strikingly, among the 26 potential regulators of hair follicle morphogenesis identified, *Arhgef3* was the only one consistently found to be differentially expressed, showing higher levels in hair placodes compared to the interfollicular epidermis (IFE), across multiple studies [19,20,36,40–42]. More specifically, our findings revealed that cells transduced with shRNAs targeting ARHGEF3 failed to contribute to hair follicles, although their representation in the epidermis remained unchanged [36]. These results suggest that while ARHGEF3 is not essential for epidermal barrier formation, it acts as a positive regulator of hair follicle development.

Although the biological and molecular functions of ARHGEF3 remain incompletely defined, emerging evidence points to its involvement in diverse cellular processes. Biochemically, ARHGEF3 functions as a RhoGEF for RHOA and RHOB through its DH-PH domain, whose catalytic activity is regulated by phosphorylation [43–45]. Beyond its canonical GEF function, ARHGEF3 can also inhibit mTORC2-AKT signaling in a GEF-independent manner to restrict myoblast differentiation [46]. In injured muscles, it limits regeneration via a RHOA-dependent mechanism, whereas *Arhgef3*^*−/−*^ mice show enhanced repair via autophagy [47]. Using this model, it was further shown that *Arhgef3* deficiency reduces weight gain in response to a high fat diet [48]. Consistent with its context-dependent functions, an independent *Arhgef3*-null model, displayed enlarged yet functional platelets [49]. Building on these findings, we now investigate a new role for ARHGEF3 in skin development, specifically, its function in regulating hair placode budding by orchestrating cell fate and development of a radial P-cadherin gradient across placode cell-cell junctions.

## Results

### ARHGEF3 is expressed in the developing skin

As previously mentioned, several groups that profiled the IFE and placodes using different enrichment strategies have shown that *Arhgef3* mRNA is differentially expressed between hair placodes and the IFE during the first wave of hair placode formation [20,40–42]. Using the *Sulic and colleagues* datasets, we performed an isoform switch analysis and found that *Arhgef3* transcript variant 3, (*ENSMUST000000224981.2; NM_001289687.1*) is the most highly expressed isoform in the placode and surrounding epidermis (S1 Fig). In situ hybridization (ISH) on E18.5 skin sections revealed that *Arhgef3* is still expressed in placodes from later waves (**Fig 1B**), and quantification revealed that its expression is significantly higher in the placode compared to the epidermis (**Fig 1C**). We also observed that *Arhgef3* is present in the hair peg. Moreover, its expression is also detectable in other skin compartments, indicating broader expression across the tissue (**Fig 1D**). Nevertheless, its upregulation in the placode at the onset of hair follicle development supports a functional role in this process.

### ARHGEF3 is not required for skin barrier formation

**Fig 2:**
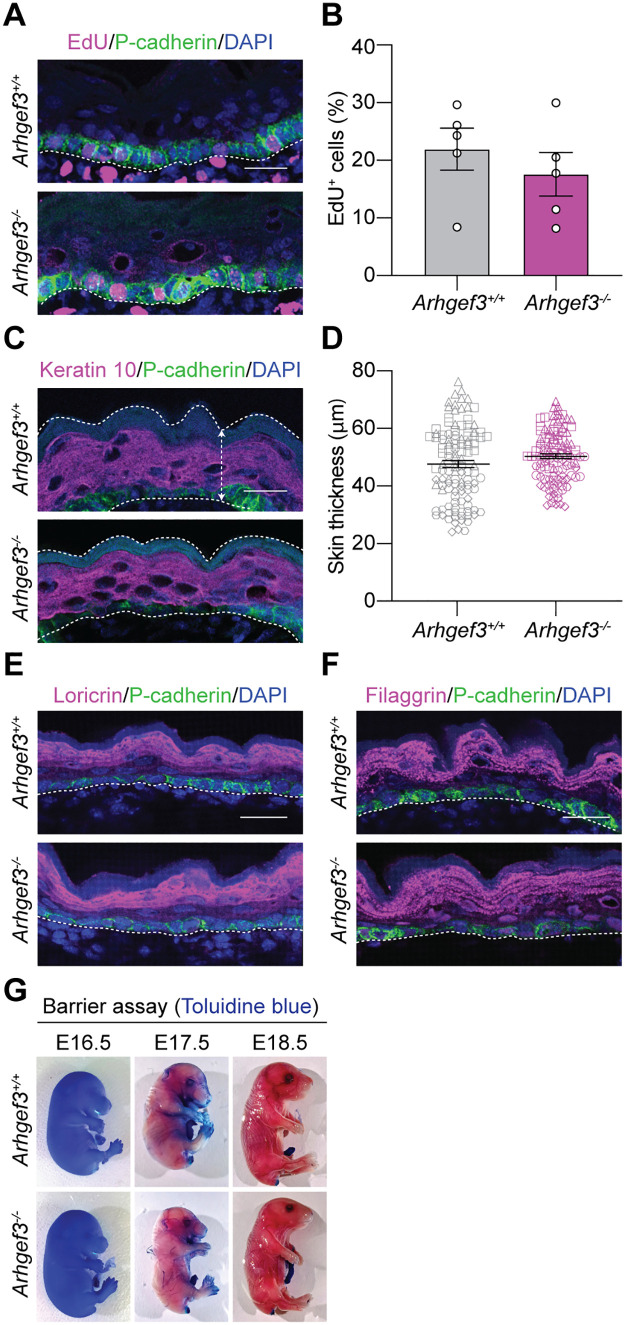
ARHGEF3 is not required for skin barrier formation. **(A)** EdU and P-cadherin immunofluorescence on E18.5 sagittal back skin sections. **(B)** Graph displays the quantification of the average percentage of basal (P-cadherin^+^) EdU^+^ cells as mean ± SEM. Statistical analyses were performed using a two-tailed Mann–Whitney test, *P* value = 0.4206, *n* = 5 embryos per genotype and the experiment was independently performed 5 times. **(C)** P-cadherin and Keratin 10 immunofluorescence on E18.5 sagittal back skin sections. **(D)** Graph displays the average skin thickness per embryo (to better display the variability in skin thickness per embryo; the data from each embryo is represented using a different symbol) as mean ± SEM. Statistical analyses were performed using a two-tailed nested *t* test, *P* value = 0.5489, *n* = 5 embryos (25, 30, 30, 15, and 15 measures) per genotype and the experiment was independently performed 5 times. **(E)** Loricrin, **(F)** Filaggrin and P-cadherin immunofluorescence on E18.5 sagittal back skin sections. Representation of *n* = 4 embryos. Proper expression of Loricrin and Filaggrin is observed in both *Arhgef3*^+*/*+^ and *Arhgef3*^*−/−*^ animals. **(G)** Barrier assays on E16.5, E17.5 and E18.5 *Arhgef3*^+*/*+^ and *Arhgef3*^*−/−*^ mouse embryos. Representation of *n* = 2 independent experiments. Scale bars: 25 µm. DAPI is used to label cell nuclei. Dotted line is used to delimit the epidermis and hair follicle from the dermis. Underlying dataset for this figure is available at: https://doi.org/10.5281/zenodo.17400745.

To investigate the role of ARHGEF3 during skin development, we used an *Arhgef3*-knockout mouse strain (*Arhgef3*^*−/−*^), generated by deleting part of exon 3, first exon shared by all four *Arhgef3* isoforms in mice [47]. These mice are viable, fertile, and exhibit enhanced muscle regeneration after injury, but their skin phenotype has not yet been characterized [47]. While our RNAi-mediated in vivo screen suggested that ARHGEF3 is dispensable for epidermal development, shRNA may only achieve partial knockdown. Given that ARHGEF3 regulates proliferation in other cell types [50,51], we examined whether its complete loss in knockout embryos affects epidermal proliferation. To do so, pregnant females were pulsed with 5-ethynyl-2′-deoxyuridine (EdU) to label cells in S-phase. Quantification of EdU^+^ basal cells, marked by P-cadherin, showed a slight but statistically non-significant reduction in *Arhgef3*^*−/−*^ embryos (**Fig 2A and 2B**). Epidermal thickness, assessed by P-cadherin immunofluorescence to delineate the epidermal base, was also unchanged (**Fig 2C and 2D**). Immunofluorescence further revealed comparable levels of Keratin 10 between genotypes, indicating normal differentiation of the suprabasal layer (**Fig 2C**). Similarly, the expression of Loricrin and Filaggrin in the granular layer of the epidermis was unaffected in *Arhgef3*^*−/−*^ embryos (**Fig 2E and 2F**). Finally, a skin barrier assay performed between E16.5 to E18.5 showed normal barrier formation in both wild-type and knockout embryos (**Fig 2G**). Collectively, these results demonstrate that ARHGEF3 is not essential for epidermal development, supporting the conclusions of our RNA-mediated in vivo screen [36].

### ARHGEF3 is required for hair follicle asymmetric downgrowth

**Fig 3:**
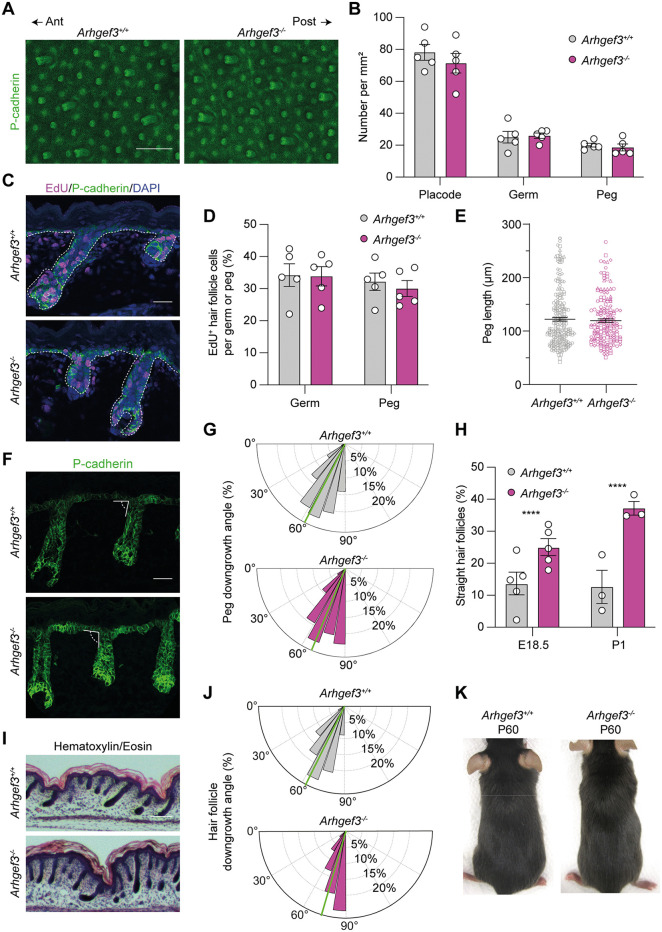
ARHGEF3 is required for hair follicle asymmetric downgrowth. **(A)** Maximum intensity Z-projections of P-cadherin whole-mount immunofluorescence of E16.5 back skin. Scale bar: 100 µm. The anterior–posterior axis of the embryo is indicated. **(B)** Graph displays the average number of hair placodes, germs, and pegs per embryo in 1 mm^2^ region of back skin as mean ± SEM. Statistical analyses were performed using two-way ANOVA followed by multiple comparisons (with Sidak’s correction) between *Arhgef3*^+*/*+^ and *Arhgef3*^*−/−*^ conditions for each hair follicle stage. All comparisons were not significant (adjusted *P* values: placode = 0.5052; germ = 0.9967; peg = 0.9911), *n* = 5 embryos per genotype and the experiment was independently performed 5 times. **(C)** EdU and P-cadherin immunofluorescence of E18.5 sagittal back skin sections. Scale bar: 25 µm. DAPI is used to label cell nuclei. Dotted white line is used to delimit the epidermis and hair follicles from the dermis. **(D)** Graph displays the average percentage of EdU^+^ hair germ and hair peg cells per embryo as mean ± SEM. Statistical analyses were performed using two-tailed Mann–Whitney tests for either germ or peg, *P* values respectively >0.9999 and 0.8413, *n* = 5 embryos per genotype and the experiment was independently performed 5 times (germ analyzed: *Arhgef3*^+*/*+^ = 91; *Arhgef3*^*−/−*^ = 83, peg analyzed: *Arhgef3*^+*/*+^ = 165; *Arhgef3*^*−/−*^ = 122). **(E)** Graph displays the average length of hair pegs in E18.5 embryo (to better display the distribution of hair peg length per embryo; the data from each embryo is represented using a different symbol) as mean ± SEM. Statistical analyses were performed using a two-tailed nested t *t*est, *P* value = 0.6359, *n* = 5 embryos per genotype and the experiment was independently performed 5 times (peg analyzed: *Arhgef3*^+*/*+^ = 23, 37, 19, 65, 71; *Arhgef3*^*−/−*^ = 34, 39, 25, 52, 42). **(F)** P-cadherin immunofluorescence of E18.5 sagittal back skin. Scale bar: 25 µm. **(G)** Rose plot displays the frequency of hair peg angle calculated in **F** with 10° bins. Green line points out the circular mean (*Arhgef3*^+*/*+^ = 62.77°; *Arhgef3*^*−/−*^ = 67.40°). Statistical analyses to compare the distribution of angles were performed using Watson *U*^2^ test for homogeneity of angles. This test reported *P* value between 0.001 and 0.01 and an approximate *P* value (after 10,000 permutations) of 0.0095, *n* = 5 embryos per genotype and the experiment was independently performed 5 times (total hair follicles analyzed: *Arhgef3*^+*/*+^ = 549; *Arhgef3*^*−/−*^ = 545). **(H)** Average percentage of straight hair pegs at E18.5 and hair follicles at P1 calculated in **F** and **I**, as mean ± SEM. Statistical analyses were performed using two-sided Fisher’s exact tests for both E18.5 and P1, *P* values <0.0001, *n* = 5 embryos (E18.5) and 3 embryos (P1) per genotype and the experiment was independently performed 5 times (E18.5) and 3 times (P1). **(I)** Hematoxylin and eosin staining of sagittal back skin at P1 from *Arhgef3*^+*/*+^ and *Arhgef3*^*−/−*^*.* Scale bar: 1,000 µm. **(J)** Rose plots display the frequency of hair follicles angle calculated in **I** with 10° bins. Green line points out the circular mean (*Arhgef3*^+*/*+^ = 63.64°; *Arhgef3*^*−/−*^ = 73.60°). Statistical analyses to compare the distribution of angles were performed using Watson *U*^2^ test for homogeneity of angles. This test reported a *P* value < 0.001 and an approximate *P* value (after 10,000 permutations) of 0.00001, *n* = 3 embryos per genotype and the experiment was independently performed 3 times (total hair follicles analyzed: *Arhgef3*^+*/*+^ = 308; *Arhgef3*^*−/−*^ = 278). **(K)** Representative pictures of *Arhgef3*^+*/*+^ and *Arhgef3*^*−/−*^ animals at P60. Underlying dataset for this figure is available at: https://doi.org/10.5281/zenodo.17400745.

With the confirmation that disrupting ARHGEF3 expression does not result in widespread epidermal defects, we proceeded to investigate the hypothesis that this RhoGEF is essential for proper hair follicle morphogenesis, as suggested by the screen, and sought to determine at which step it is playing a key role [36]. First, we assessed whether ARHGEF3 is required for hair follicle formation. To this end, we used whole-mount immunofluorescence of P-cadherin on E16.5 back skin, which allowed us to visualize hair placodes, germs, and pegs from the staggered waves of hair follicle development (**Figs 1A and 3A**). Quantitative analysis of these structures showed no significant difference in their average number between control and *Arhgef3*-null samples (**Fig 3B**). To determine whether the complete loss of ARHGEF3 affects cell proliferation in the hair follicle, we performed an EdU pulse experiment (**Fig 3C**). Quantification of EdU^+^ cells in hair germs and pegs from E18.5 back skin revealed no significant differences between *Arhgef3*^+*/*+^ and *Arhgef3*^*−/−*^ animals (**Fig 3D**). This conclusion was further supported by the quantification of average hair peg length, which showed no significant difference between control and *Arhgef3*^*−/−*^ embryos (**Fig 3E**). Altogether, these results emphasize that ARHGEF3 is not essential for hair follicle formation per se in the skin.

As hair follicles develop in mice, they typically align along the anterior-posterior axis of the embryo, a pattern observed in both *Arhgef3*^+*/*+^ and *Arhgef3*^*−/−*^ embryos (**Fig 3A**). In addition, hair follicles penetrate the dermis asymmetrically, entering at an angle relative to the basement membrane rather than perpendicularly [24]. Intriguingly, our analysis of embryonic skin at E18.5 revealed that this process is disrupted in the absence of ARHGEF3, as evidenced by the frequent observation of hair pegs lacking this characteristic asymmetry (**Fig 3F**). In wild-type embryos, hair follicles extended into the dermis at an average angle of 63° relative to the basement membrane (**Fig 3G**, green line in the top graph). By contrast, *Arhgef3*-null hair follicles showed a statistically significant shift in angular distribution, with an increased average angle of 67° (**Fig 3G**; green line in the bottom graph). More strikingly, we observed a marked increase in the proportion of perpendicular (straight) hair pegs in *Arhgef3*^*−/−*^ embryos compared to the wild-type animals at E18.5 (**Fig 3H**). While the percentage of straight hair follicles remained close to 10% in *Arhgef3*^+*/*+^ newborn (P1: postnatal day 1), it increased to 37% in knockout animals, indicating that all hair follicle waves are affected by the absence of ARHGEF3 (**Fig 3H and 3I**). Hair follicles that were properly tilted were also consistently aligned along the anterior-posterior axis of the pups (**Fig 3I and 3J**). Interestingly, the presence of these angled hair follicles likely conceals the presence of perpendicular ones, as *Arhgef3*^+*/*+^ and *Arhgef3*^*−/−*^ animals appear phenotypically indistinguishable to the naked eye at P60 (**Fig 3K**).

**Fig 4:**
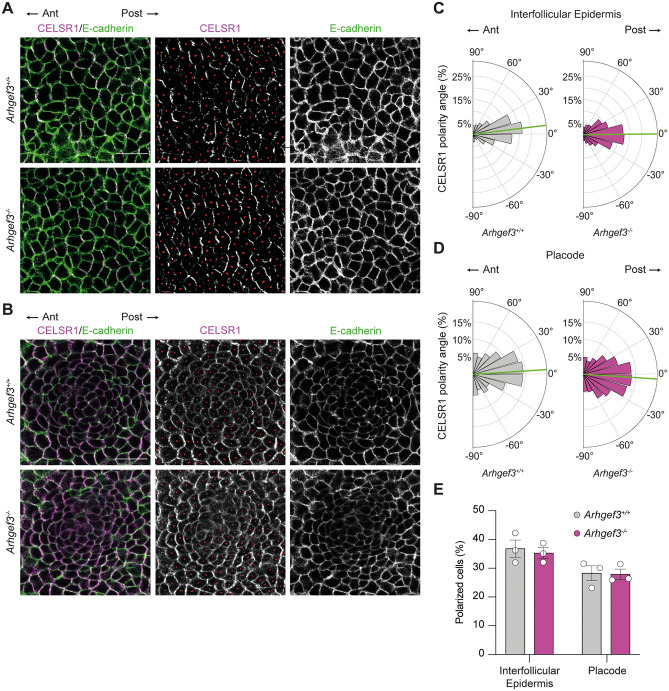
ARHGEF3 is not required to establish CELSR1 planar polarized domain in the epidermis. **(A, B)** CELSR1 and E-cadherin whole-mount immunofluorescence of E15.5 back skin focused on (A) IFE and (B) early placode. Scale bars: 25 µm. The anterior–posterior axis of the embryo is indicated. **(C, D)** Rose plots display the orientation of the opposing CELSR1 domains in the IFE (C) and placode cells (D), 0° being perfectly aligned with the anterior-posterior axis of the tissue (bins: 15°). Green lines point out the circular means (IFE *Arhgef3*^+*/*+^ = 7.09°; *Arhgef3*^*−/−*^ = 0.55°; placode *Arhgef3*^+*/*+^ = 3.74°; *Arhgef3*^*−/−*^ = −3.76°). Statistical analyses to compare the distribution of angles were performed using Watson *U*^2^ test for homogeneity of angles. This test reported approximate *P* values (after 10,000 permutations) of 0.0002 and <0.0001 (IFE and placode cells, respectively), *n* = 3 embryos per genotype and the experiment was independently performed 3 times (total cells analyzed: IFE *Arhgef3*^+*/*+^ = 641, *Arhgef3*^*−/−*^ = 724; placode *Arhgef3*^+*/*+^ = 1,394, *Arhgef3*^*−/−*^ = 1,115). **(E)** Average percentage of polarized cells in IFE and placodes calculated in A and B, as mean±SEM. Statistical analyses were performed using two-sided Fisher’s exact tests for both IFE and placode cells, *P* values = 0.2589 and 0.6873, *n* = 3 embryos per genotype and the experiment was independently performed 3 times. Underlying dataset for this figure is available at: https://doi.org/10.5281/zenodo.17400745.

Among the mechanisms that regulate hair follicle polarization and alignment in mice is PCP, which refers to the coordinated polarization of cells within the plane of an epithelium. During skin development, the progressive partitioning of core PCP components, such as CELSR1, in basal epidermal and early placode cells provides instructive cues that guide hair follicle orientation [24]. To determine whether PCP is properly established in the absence of ARHGEF3, we performed whole-mount immunofluorescence for CELSR1 and E-cadherin on E15.5 back skin tissue, examining both the epidermis and developing placode (**Fig 4A and 4B**). Quantification of the angle of CELSR1 domains revealed a consistent polarization along the anterior–posterior axis of the embryos (180°–0°) in the IFE (**Fig 4C**) as well as in the developing placodes (**Fig 4D**). Although there is a variation in the overall distribution of those angles around the expected axis, more pronounced in the placode, a similar percentage of cells showed appropriate alignment along the anterior-posterior axis in both compartments and genotypes (**Fig 4E**). These findings suggest that CELSR1 polarization is properly established in the epidermis of *Arhgef3-null* embryos, indicating that the hair follicle angling defects observed in the absence of ARHGEF3 are likely uncoupled from the initial partitioning of CELSR1 in the establishment of PCP in the epidermis.

### ARHGEF3 restricts radial cell fate patterning in the placode

**Fig 5:**
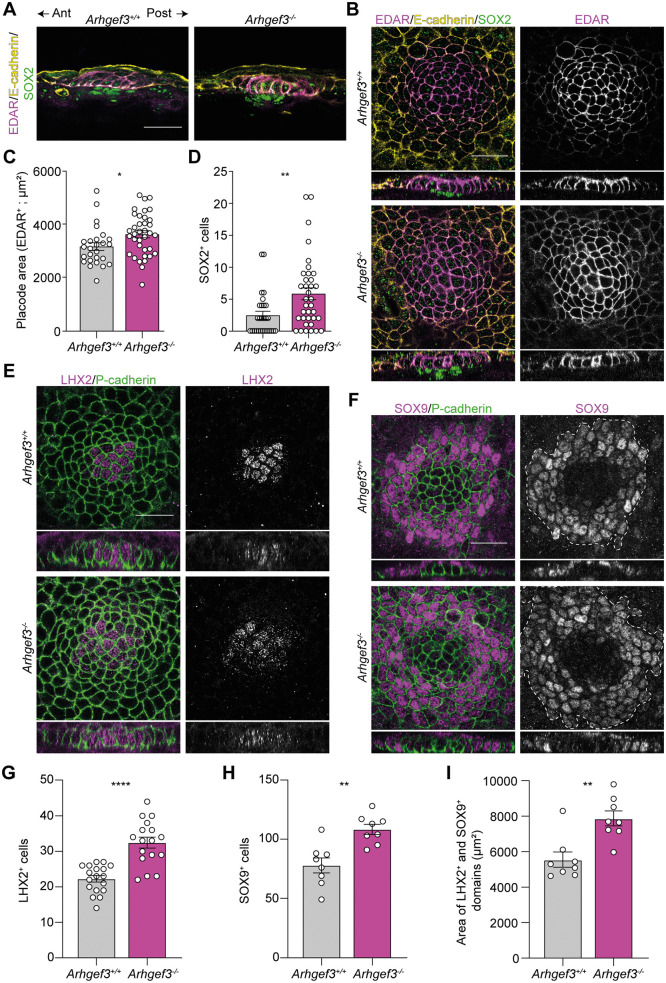
ARHGEF3 restricts placode cell fate acquisition. **(A)** EDAR, E-cadherin, and SOX2 immunofluorescence of E15.5 sagittal back skin sections. The anterior–posterior axis of the embryo is indicated. **(B)** EDAR, E-cadherin, and SOX2 whole-mount immunofluorescence of E15.5 hair placodes with orthogonal XZ views below. **(C)** Graph displays the average area of EDAR^+^ domain as mean ± SEM. Statistical analyses were performed using a two-tailed Mann–Whitney test, *P* value = 0.0102, *n* = 4 embryos per genotype and the experiment was independently performed 4 times (total placode analyzed: *Arhgef3*^+*/*+^ = 28, *Arhgef3*^*−/−*^ = 36). **(D)** Graph displays the average number of SOX2^+^ cells below the EDAR^+^ domain as mean ± SEM. Statistical analyses were performed using a two-tailed Mann–Whitney test, *P* value = 0.0035, *n* = 4 embryos per genotype and the experiment was independently performed 4 times (total placode analyzed: *Arhgef3*^+*/*+^ = 28, *Arhgef3*^*−/−*^ = 36). **(E)** LHX2 and P-cadherin whole-mount immunofluorescence of E15.5 hair placodes with orthogonal XZ views below. **(F)** SOX9 and P-cadherin whole-mount immunofluorescence of E15.5 hair placodes with orthogonal XZ views below. Dashed line delimits the SOX9^+^ area. **(G)** Graph displays the average number of LHX2^+^ cells per placode as mean ± SEM. Statistical analyses were performed using a two-tailed Mann–Whitney test, *P* value < 0.0001, *n* = 3 embryos per genotype and the experiment was independently performed 3 times (total placode analyzed: *Arhgef3*^+*/*+^ = 18, *Arhgef3*^*−/−*^ = 17). **(H)** Graph displays the average number of basal SOX9^+^ cells per placode as mean ± SEM. Statistical analyses were performed using a two-tailed Mann–Whitney test, *P* value = 0.0019, *n* = 5 embryos per genotype and the experiment was independently performed 5 times (total placode analyzed: *Arhgef3*^+*/*+^ = 8, *Arhgef3*^*−/−*^ = 8). **(I)** Graph displays the average area of LHX2^+^ and SOX9^+^ domains as mean±SEM. Statistical analyses were performed using a two-tailed Mann–Whitney test, *P* value = 0.0047, *n* = 5 embryos per genotype and the experiment was independently performed 5 times (total placodes analyzed: *Arhgef3*^+*/*+^ = 8, *Arhgef3*^*−/−*^ = 8). Scale bars: 25 µm. Underlying dataset for this figure is available at: https://doi.org/10.5281/zenodo.17400745.

Since the asymmetric downgrowth defect observed in *Arhgef3*^*−/−*^ embryos is not caused by a general disruption of PCP establishment, we next investigated whether ARHGEF3 contributes to epidermal placode cell fate specification, polarization, and budding. Among the earliest molecular markers of placode formation are EDAR expression within the placode epithelium and SOX2 activation in the underlying fibroblasts destined to form the dermal condensate [6]. In both *Arhgef3*^+/+^ and *Arhgef3*^*−/−*^ embryos, an EDAR^+^ epithelial domain and an underlying SOX2^+^ dermal condensate were observed (**Fig 5A and 5B**). However, in the absence of ARHGEF3, the EDAR^+^ domain was significantly enlarged compared to wild-type controls (**Fig 5C**). This increase in placode size was accompanied by a proportional expansion of SOX2^+^ dermal condensate cells in *Arhgef3*-null embryos (**Fig 5D**).

We then assessed whether radial cell fate patterning was properly established in the absence of ARHGEF3. Previous studies have shown that early hair placodes contain a central cluster of LHX2-expressing cells [19], surrounded by an outer ring of SOX9^+^ cells that forms prior to placode budding [4]. To determine whether cell fate specification depends on ARHGEF3, we performed whole-mount immunofluorescence for LHX2 and SOX9 on E15.5 back skin (**Fig 5E and 5F**). In placodes from both *Arhgef3*^+/+^ and *Arhgef3*^*−/−*^ embryos, a central LHX2^+^ cell population was observed (**Fig 5E**). However, in the absence of ARHGEF3, this LHX2^+^ domain was significantly expanded (**Fig 5G**), indicating that ARHGEF3 acts to restrict the size of the LHX2^+^ compartment. SOX9^+^ cells were also present in both genotypes (**Fig 5F**), but their number was significantly increased in *Arhgef3*^*−/−*^ embryos, mirroring the expansion of the EDAR^+^ and LHX2^+^ populations (**Fig 5H**). These findings reveal that although radial patterning is preserved in the absence of ARHGEF3, the domains of both LHX2^+^ and SOX9^+^ cells are expanded (**Fig 5I**).

**Fig 6:**
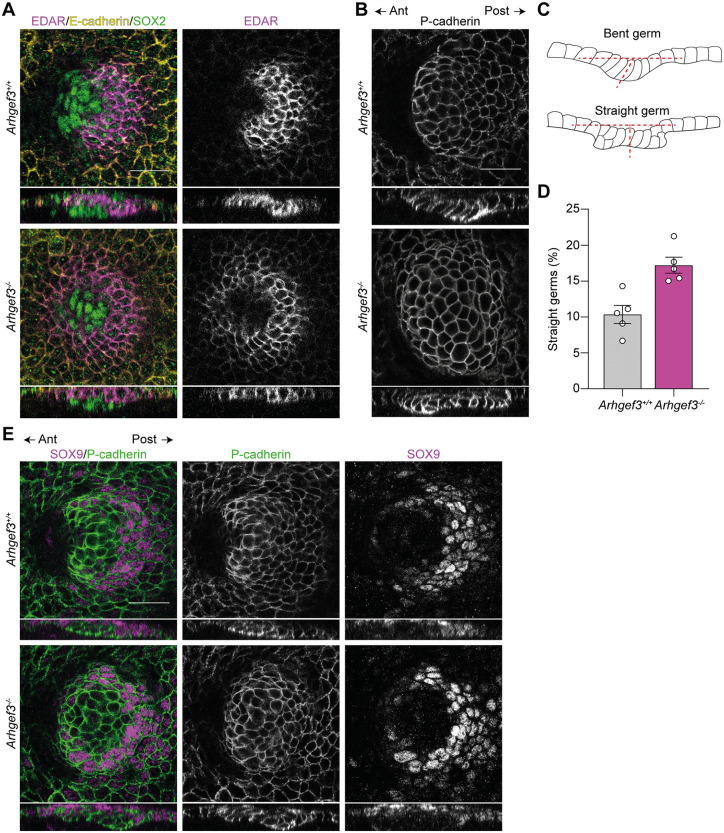
ARHGEF3 coordinates hair germ downgrowth. **(A)** EDAR, E-cadherin and SOX2 whole-mount immunofluorescence of E15.5 hair germs, with orthogonal XZ views below showing the position of the dermal condensate. **(B)** P-cadherin whole-mount immunofluorescence of E15.5 back skin showing a correctly angled (*Arhgef3*^+*/*+^*)* and straight (*Arhgef3*^*−/−*^*)* germ. The anterior–posterior axis of the embryo is indicated. **(C)** Schematic representation of angled and straight hair follicle development as seen from the orthogonal view of whole-mount germs. **(D)** Average percentage of straight germs at E16.5 as mean ± SEM. Statistical analyses were performed using two-sided Fisher’s exact test, *P* value = 0.1354, *n* = 5 embryos per genotype and the experiment was independently performed 5 times (total germ analyzed: *Arhgef3*^+*/*+^ = 106, *Arhgef3*^*−/−*^ = 126). **(E)** SOX9 and P-cadherin whole-mount immunofluorescence of E15.5 back skin showing proper polarization of SOX9^+^ cells in both genotypes, despite the absence of tilt in the *Arhgef3*^*−/−*^ hair bud. Scale bars: 25 µm. Underlying dataset for this figure is available at: https://doi.org/10.5281/zenodo.17400745.

As development progressed, cellular rearrangements lead to the formation of anteriorly tilted hair germs, which are associated with a dermal condensate positioned at the anterior tip of the developing hair follicle. Using EDAR and SOX2 immunofluorescence to mark the hair germ and dermal condensate, respectively, this characteristic patterning was clearly observed in the skin of *Arhgef3*^+*/*+^ controls (**Fig 6A**). In contrast, *Arhgef3*^*−/−*^ embryos frequently failed to undergo this anterior tilting, instead forming perpendicular epithelial extensions accompanied by a vertically aligned dermal condensate (**Fig 6A**). The presence of straight hair germs in *Arhgef3*^*−/−*^ embryos was further confirmed by P-cadherin staining and illustrated in the accompanying schematic. (**Fig 6B and 6C**). We observed a 1.66-fold increase in the frequency of straight hair germs in the absence of ARHGEF3 (**Fig 6D**)*.* Although this difference did not reach statistical significance, it suggests a clear trend that may contribute to the hair follicle angling defect observed (**Fig 3H**). In control embryos, hair germs tilting was associated with the posterior relocalization of SOX9^+^ cells (**Fig 6E**) [2,4]. Interestingly, in *Arhgef3*^*−/−*^ placodes, SOX9^+^ cells were still correctly repositioned to the posterior pole despite the absence of asymmetric tilting, suggesting that SOX9 polarization can occur independently of directional downgrowth.

### ARHGEF3 regulates P-cadherin patterning in the skin

**Fig 7:**
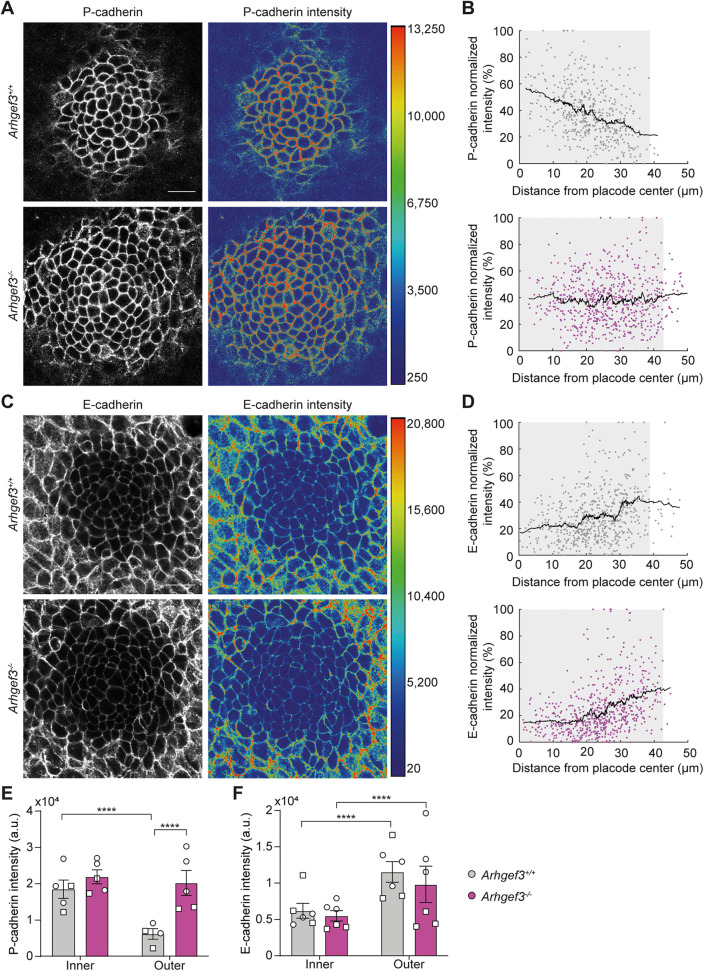
ARHGEF3 is required for the establishment of a P-cadherin radial gradient. **(A)** P-cadherin whole-mount immunofluorescence of E15.5 hair placodes. The right panel shows P-cadherin intensity in pseudocolor with the corresponding scale to the right side. **(B)** Graphs display normalized P-cadherin mean intensity at cell-cell junction according to cell position relative to placode center in *Arhgef3*^+*/*+^ (top panel) and *Arhgef3*^*−/−*^ (bottom panel) embryos. Each point represents a cell; black lines point the trend for *n* = 5 placodes from 2 embryos per genotype and the experiment was independently performed 2 times (total cells analyzed: *Arhgef3*^+*/*+^ = 497 *Arhgef3*^*−/−*^ = 630). Gray shadow represents the average radius of analyzed placodes. **(C)** E-cadherin whole-mount immunofluorescence of E15.5 hair placodes. The right panel shows E-cadherin intensity in pseudocolor with the corresponding scale to the right side. Scale bars: 25 µm. **(D)** Graphs display normalized mean E-cadherin intensity at cell-cell junction according to cell position relative to placode center in *Arhgef3*^+*/*+^ (top panel) and *Arhgef3*^*−/−*^ (bottom panel) embryo. Each point represents a cell, black lines point the trend for *n* = 6 placodes from 2 embryos per genotype and the experiment was independently performed 2 times (total cells analyzed: *Arhgef3*^+*/*+^ = 561, *Arhgef3*^*−/−*^ = 636). Gray shadow represents the average radius of analyzed placodes. **(E)** Graph displays P-cadherin intensity (a.u.) at cell junction in the inner (0-10 µm) and outer (30–40 µm) cells of the placode as mean±SEM. Statistical analyses were performed using a Kruskal–Wallis test followed by Dunn’s multiple comparisons with *n* = 5 placodes from 2 embryos (represented by circle and square symbols) per genotype and the experiment was independently performed 2 times (total cells analyzed: *Arhgef3*^+*/*+^ = 50 (inner) and 29 (outer), *Arhgef3*^*−/−*^ = 31 (inner) and 174 (outer)). Adjusted *P* values are: *Arhgef3*^+*/*+^ inner vs. outer <0.0001; *Arhgef3*^*−/−*^ inner vs. outer = 0.5928; *Arhgef3*^+*/*+^ inner vs. *Arhgef3*^*−/−*^ inner = 0.2044; *Arhgef3*^+*/*+^ outer vs. *Arhgef3*^*−/−*^ outer < 0.0001. **(F)** Graph displays E-cadherin intensity (a.u.) at cell junction in the inner (0–10 µm) and outer (30–40 µm) cells of the placode as mean ± SEM. Statistical analyses were performed using a Kruskal–Wallis test followed by Dunn’s multiple comparisons with *n* = 6 placodes from 2 embryos (represented by circle and square symbols) per genotype and the experiment was independently performed 2 times (total cells analyzed: *Arhgef3*^+*/*+^ = 66 (inner) and 86 (outer), *Arhgef3*^*−/−*^ = 53 (inner) and 154 (outer)). Adjusted *P* values are: *Arhgef3*^+*/*+^ inner vs. outer <0.0001; *Arhgef3*^*−/−*^ inner vs. outer <0.0001; *Arhgef3*^+*/*+^ inner vs. *Arhgef3*^*−/−*^ inner >0.9999; *Arhgef3*^+*/*+^ outer vs. *Arhgef3*^*−/−*^ outer > 0.9999. Underlying dataset for this figure is available at: https://doi.org/10.5281/zenodo.17400745.

Previous work has shown that straight but polarized hair germs can also form in *E-cadherin* cKO embryos. In this context, a compensatory upregulation of P-cadherin correlates with delayed cellular rearrangements and a failure to establish a proper radial P-cadherin gradient [4]. We asked whether a similar mechanism might underlie the straight hair follicle phenotype observed in *Arhgef3*^*−/−*^ embryos. To investigate this, we performed whole-mount immunofluorescence for E- and P-cadherin on E15.5 back skin and quantified their junctional intensity across the placode. As expected, control placodes displayed opposing radial gradients, where P-cadherin expression was the highest at the center and progressively decreased toward the periphery, while E-cadherin expression increased toward the periphery (Fig 7A–7D). In *Arhgef3*^*−/−*^ placodes, the E-cadherin gradient remained intact; however, the radial distribution of P-cadherin was disrupted (Fig 7A–7D).

Quantification revealed that P-cadherin levels at cell-cell junctions were significantly higher in the outer region of *Arhgef3*^*−/−*^ placodes, where levels failed to decrease as expected (**Fig 7E**). In contrast, E-cadherin levels at both central and peripheral junctions were comparable between genotypes (**Fig 7F**). This increase in P-cadherin was not restricted to the placodes, as we also observed elevated P-cadherin levels at cell-cell junctions within the IFE, although the fold change between *Arhgef3*^+*/*+^ and *Arhgef3*^*−/−*^ was less pronounced than in the outer placode region. Meanwhile, E-cadherin levels remained unaffected across the tissue (S2 Fig). Together, these findings suggest that ARHGEF3 acts upstream of P-cadherin distribution during skin development, promoting the establishment of a radial P-cadherin gradient that is essential for asymmetric placode budding.

### ARHGEF3 promotes F-actin accumulation at cell-cell junctions

**Fig 8:**
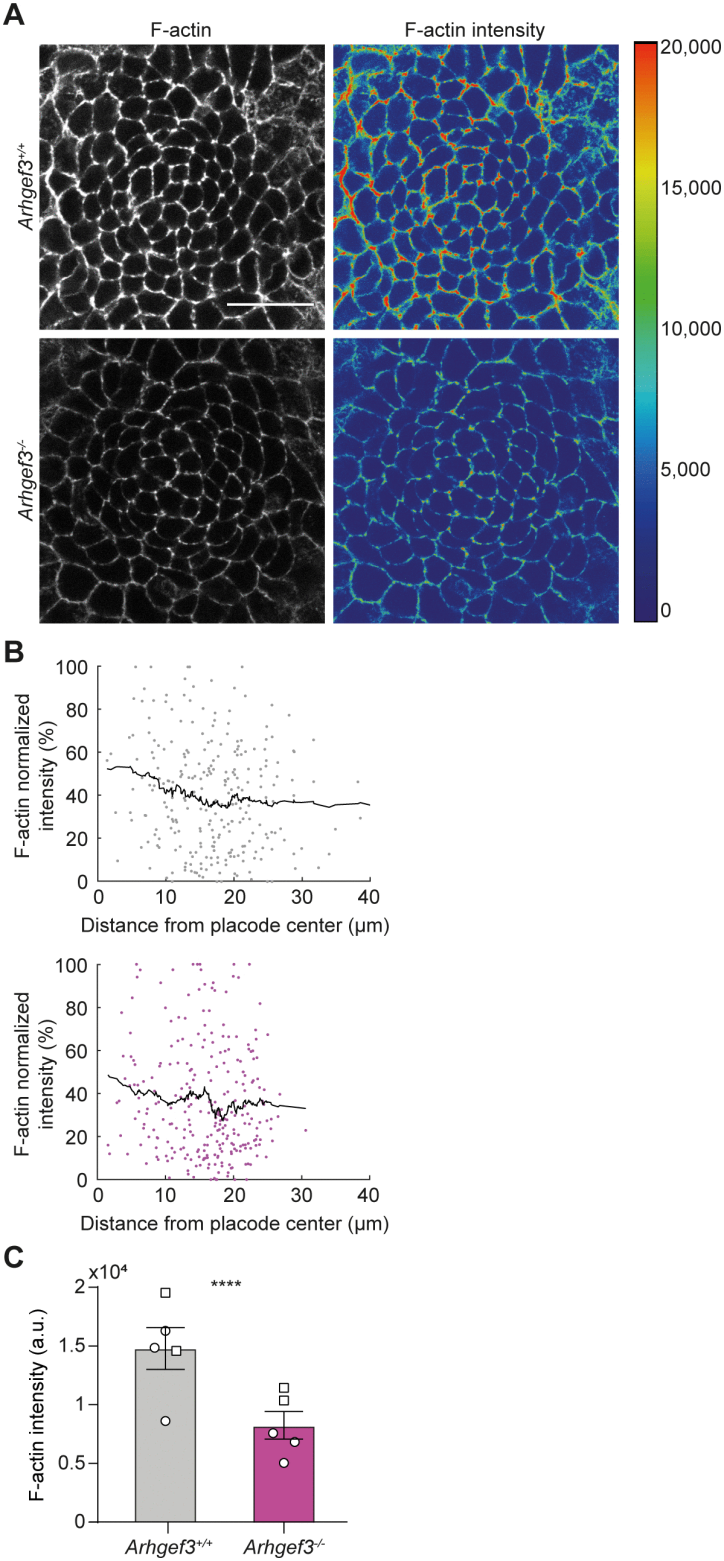
ARHGEF3 controls junctional F-actin levels in placodes. **(A)** F-actin whole-mount immunofluorescence of E15.5 hair placodes. The right panel shows F-actin intensity in pseudocolor with the corresponding scale to the right side. Scale bar: 25 µm. **(B)** Graphs display normalized mean F-actin intensity at cell-cell junction relative to placode center in *Arhgef3*^+*/*+^ (top panel) and *Arhgef3*^*−/−*^ (bottom panel) embryos. Each point represents a cell; black lines point the trend for *n* = 5 placodes from 2 embryos per genotype and the experiment was independently performed 2 times. **(C)** Graph displays F-actin intensity (a.u.) at cell junction in the cells of the placode as mean ± SEM. Statistical analyses were performed using a two-tailed Mann–Whitney test, *P* value<0.0001, *n* = 5 placodes from 2 embryos (represented by circle and square symbols) per genotype and the experiment was independently performed 2 times (total cells analyzed: *Arhgef3*^+*/*+^ = 263, *Arhgef3*^*−/−*^ = 269). Underlying dataset for this figure is available at: https://doi.org/10.5281/zenodo.17400745.

Given the known role of ARHGEF3 as a RHO-specific GEF, we next investigated whether the phenotypes observed in *Arhgef3*^*−/−*^ placodes were also associated with defects in actin cytoskeleton organization. To address this, we performed whole-mount immunofluorescence for F-actin on E15.5 back skin and quantified its levels at cell-cell junctions (**Fig 8A**). Although the overall pattern of F-actin distribution in developing placodes appeared similar between *Arhgef3*^+*/*+^ and *Arhgef3*^*−/−*^ embryos (**Fig 8B**), the intensity of junctional F-actin was significantly reduced in the absence of ARHGEF3 (**Fig 8C**). A comparable reduction in junctional F-actin was also observed in the IFE, where *Arhgef3*^*−/−*^ embryos displayed lower F-actin levels than controls (S3 Fig). Together, these findings identify ARHGEF3 as a key RhoGEF that promotes F-actin accumulation at cell-cell junctions in the epidermis, while also acting to restrict P-cadherin levels, likely contributing to the regulation of interfacial tension within the epidermis and developing placodes.

## Discussion

Building on an in vivo morphogenesis screen and transcriptomic datasets, showing elevated *Arhgef3* expression in hair placodes compared to the IFE [20,36,41], our study identifies ARHGEF3 as a novel regulator of asymmetric hair follicle downgrowth. Specifically, ARHGEF3 acts upstream to coordinate the organization of F-actin and P-cadherin at cell-cell junctions. The phenotype observed in *Arhgef3*^−*/*−^ embryos recapitulates some features of *E-cadherin* cKO; in both contexts, P-cadherin is abnormally elevated in the IFE and within placodes, disrupting the normal radial gradient that decreases outward from the placode center [4,32]. In the *E-cadherin* mutants, this disruption correlates with a failure to execute cellular rearrangements required for timely polarization. As a result, many placodes initiate symmetrical, vertically oriented budding, with some resolving their asymmetry only after invading the dermis. Although E-cadherin expression remains intact in *Arhgef3* mutants, we hypothesize that the combined effects of altered P-cadherin and reduced cortical F-actin compromise the establishment of differential interfacial tension, likely mediated by distinct cytoskeletal effectors recruited by E- versus P-cadherin. This imbalance may delay or destabilize the morphogenetic choreography required for asymmetric downgrowth. Variation in the timing and extent of these rearrangements may underlie the heterogeneity in follicle orientation observed in *Arhgef3*^*−/−*^ animals. While *P-cadherin* knockout models have not been directly assessed for placode polarization defects, studies have reported increased E-cadherin expression in mutant hair follicles, suggesting that elevated E-cadherin may similarly disrupt timely cell rearrangements, an intriguing possibility that remains to be determined [33]. Understanding how ARHGEF3 selectively restricts P-cadherin at cell-cell junctions while maintaining stable E-cadherin levels represents an exciting avenue for future research. As the ARHGEF3 interactome remains poorly defined, identifying its binding partners could uncover both GEF-dependent and GEF-independent mechanisms governing epithelial patterning and tissue polarity.

Intriguingly, beyond its role in regulating P-cadherin and cortical F-actin at cell-cell junctions, ARHGEF3 also functions upstream of EDAR, LHX2 and SOX9 fate specification in the skin. An increase in placode size through expansion of the EDAR^+^, LHX2^+^ or SOX9^+^ domains has not been reported in *E-cadherin* cKO embryos, suggesting that *Arhgef3*^*−/−*^ embryos do not fully recapitulate E-cadherin cKO phenotype [4,32]. Given that LHX2 expression depends on WNT signaling, one possibility is that ARHGEF3 modulates this pathway to influence cell fate. Supporting this idea, previous studies have shown that proper actin levels in the IFE are required for effective WNT signaling, and that depletion of WAVE complex components, resulting in reduced actin, can induce ectopic SOX9 expression via WNT activation [14]. In *Arhgef3*^*−/−*^ embryos, although junctional actin is diminished, we did not observe ectopic SOX9 expression in the IFE. Instead, loss of ARHGEF3 led to an increased number of LHX2^+^ and SOX9^+^ cells within placodes only, without affecting the total number of placodes formed. Interestingly, treatment of skin explants with Noggin has been shown to enhance WNT signaling [34]. Moreover, Noggin overexpression has been associated with larger placodes and with LHX2 induction in tumoral placodes, raising the possibility that ARHGEF3 may also intersect with BMP signaling [52,53]. However, how ARHGEF3 restricts the acquisition of placode cell fates remains unclear. Further investigation will be required to determine how ARHGEF3 modulates upstream fate specification cues, potentially through coordinated regulation of WNT and BMP pathways.

Further supporting a link between ARHGEF3 and developmental signaling pathways, *Arhgef3.2*, the Xenopus orthologue of *Arhgef3*, has also been implicated in the WNT/PCP pathway. During gastrulation, Arhgef3.2 coordinates convergent extension cell movements via its interaction with Dsh2 and DAAM, two components of the non-canonical WNT/PCP signaling pathway [54,55]. Similarly, in hair follicle morphogenesis, PCP proteins are crucial not only for providing instructive cues from the epidermis but also for coordinating cellular movements within the developing hair follicle [2,24]. These movements resemble convergent extension and are necessary for proper hair follicle polarization. While it remains unclear if ARHGEF3 functions downstream of Dsh2 in mammalian cells, our data suggest that ARHGEF3 is not required for the initial partitioning of CELSR1 in the skin. Nonetheless, whether it mediates the activity of other PCP components remains an open question and warrants further study.

While our research has primarily focused on ARHGEF3’s role in the hair placodes, it is important to note that hair follicle polarization and progenitor cell asymmetry also depend on dermal contributions [2,10]. Because we employed a full knockout mouse model, some of the observed phenotypes may be influenced by alterations in dermal signaling or changes in interfacial tension between the dermal and epidermal compartments. To more precisely define ARHGEF3’s role in dermal versus epidermal compartments, generating a conditional *Arhgef3* mouse model would be invaluable and could significantly enhance our understanding of its biological functions. Notably, ISH at E18.5 further highlights the broad expression of *Arhgef3* mRNA around birth, suggesting potential functions beyond early placode development in the skin, which could be further explored using a conditional mouse model.

Taken together, our findings identify ARHGEF3 as a critical regulator that restricts cell fate specification and establishes a proper P-cadherin gradient at cell-cell junctions across placodes, thereby facilitating the coordinated cellular rearrangements required for robust asymmetric placode budding. Given the central role of the placode in the formation of diverse epithelial appendages, future studies will be essential to explore the broader functions of ARHGEF3 in epithelial morphogenesis and tissue patterning.

## Materials and methods

### Ethics statement

We certify that all mouse experiments were conducted in strict accordance with the principles enunciated by the Canadian Council on Animal Care and the Université Laval institutional policy (Protocol Number: [2021-692, CHU-21-692]). All efforts were made to minimize animal suffering.

### Animals models

*Arhgef3*^*−/−*^ mice were previously generated and described [39]. Animals were rederived upon their arrival at the CHU de Québec—Université Laval research center, where they are now maintained in a mouse-specific pathogen-free (SPF) facility. All mouse experiments were approved by Université Laval Animal Care Protection Committee, and they followed the Canadian Council of Animal Care Guidelines.

### RNA-Seq analysis

Whole-genome RNA-sequencing data of a total of 7 placode-enriched and 7 IFE samples from E14.5 mice was collected from the *Sulic and colleagues* (Gene Expression Omnibus accession number GSE212652). Raw fastq files were quality-checked with FastQC. Transcript-level quantification was obtained with Salmon using selective alignment against the mm39 reference genome and transcriptome [56]. Gene- and transcript-level fragments per kilobase per million (FPKMs) were extracted using the *IsoformSwitchAnalyzeR* R package and plotted using GraphPad Prism 10 [57].

### RNAscope in situ hybridization

RNAscope ISH was performed using the RNAscope Multiplex Fluorescent V2 Assay (Advanced Cell Diagnostics, 323270) according to the manufacturer’s protocol. Briefly, the back skin from E18.5 embryo was dissected and fixed with 4% paraformaldehyde (PFA) for 1 h at 4 °C, washed several times with 1× phosphate-buffered saline (PBS), and dehydrated in 20% sucrose overnight. The next day, the embryos were embedded and frozen in Tissue Plus O.C.T. Compound Clear (Fisher Scientific, 4585). Sections of 14 µm were generated, baked for 30 min at 60 °C, and fixed for 15 min at 4 °C with 4% PFA. Sections were then dehydrated with serial incubations in increasing concentrations of ethanol (50%, 70%, 100% twice), treated with H_2_O_2_ for 10 min at room temperature (RT), and with Protease IV (Advanced Cell Diagnostics, 322336) for 30 min at RT. Subsequent hybridizations (*Arhgef3* or *Polr2a* probes, 2 h at 40 °C) and amplifications (Amp1 (30 min, 40 °C), Amp2 (30 min, 40 °C), and Amp3 (15 min, 40 °C)) were alternated with washes (twice, 2 min at RT) with 1× washing buffer (Advanced Cell Diagnostics, 310091). Both probes were in the C1 channel, and fluorescence was developed using the HRP-C1 reagent, followed by TSA Vivid Fluorophore 570 (Advanced Cell Diagnostics, 323272; 1:2,000) and HRP blocker. Sections were counterstained with DAPI (Advanced Cell Diagnostics, 320858), mounted using Invitrogen ProLong Diamond Antifade Mounting media (ThermoFisher, P36970), and captured using an LSM-900 confocal microscope (Zeiss) with a LD C-Apochromat 40× water immersion objective (NA: 1.1). Basic image adjustments were performed in Fiji (ImageJ).

*To quantify Arhgef3 foci per cell,* the region of interest in the placode or IFE was outlined. In Fiji, the 3D object counter option was used to count the foci across all planes. The number of DAPI positive cells was counted manually.

### Immunofluorescence, microscopy, and image processing

For whole-mount immunofluorescence on back skin tissues, embryos were fixed for 1 h using 4% PFA at RT. The embryos were washed several times in 1× PBS while gently shaking and left to wash overnight. The following day the skin was dissected and blocked in gelatin buffer (1× PBS supplemented with 2.5% normal donkey serum (Sigma, 566460), 1% bovine serum albumin (BSA) (Wisent, 800-095-EG), 2% gelatin from cold water fish skin (Sigma, G7765) and 0.3% Triton X-100 (BioShop, TRX506.100) for at least 2 h with agitation at RT. For whole-mount CELSR1 staining, the gelatin blocking buffer contained 2.5% fish gelatin, 2.5% normal donkey serum, 2.5% normal goat serum (Sigma, NSO2L), 0.5% BSA, and 0.1% Triton X-100 in 1× PBS and all the washes were done using 0.1% Triton X-100 in 1× PBS. Primary antibodies (see below) were diluted in gelatin buffer and incubated with agitation overnight at 4 °C. The next day, the skin was washed 5 times with 0.3% Triton X-100 in 1× PBS. Secondary antibodies were diluted in gelatin buffer and incubated overnight with agitation at 4 °C. The following day, the back skin was washed with 0.3% Triton X-100 in 1× PBS for at least 3 h changing the solution at least 3 times, and then incubated with DAPI (Sigma, D9542; 0.2 µg/ml) for 20 min. The nuclear stain was washed with 1× PBS followed by dH_2_O after which tissues were mounted on slides using Invitrogen ProLong Diamond Antifade Mounting media (ThermoFisher, P36970). Primary antibodies were used as follows: P-cadherin (Cadherin-3, R&D, AF761; 1:400), E-cadherin (Cadherin-1, ThermoFisher, 14-3249-82; 1:1,000), CELSR1 (Fuchs lab gift, rabbit; 1:300), SOX9 (Millipore Sigma, AB5535; 1:1,000), LHX2 (Abcam, ab184337; 1:250), EDAR (Novus Biologicals, AF745; 1:200), and SOX2 (Cell Signaling Technology, C70B1; 1:200). Secondary antibodies were used as follows: donkey anti-goat IgG cross-adsorbed Alexa Fluor 647 (ThermoFisher, A-21447; 1:500), donkey anti-rabbit IgG cross-adsorbed Alexa Fluor 488 (ThermoFisher, A21206; 1:500), and donkey anti-rat IgG cross-adsorbed Alexa Fluor 594 (ThermoFisher, A21209; 1:500). Phalloidin Rhodamine (Thermo Fisher Scientific, R415; 1:1,000) was used to label F-actin.

For immunofluorescence on cryosections, the back skin of embryos was fixed with 4% PFA for 1 h at 4 °C, washed several times with PBS 1×, and dehydrated in 20% sucrose overnight. The next day, the skin tissues were embedded and frozen in Tissue Plus O.C.T. Compound Clear (Fisher Scientific, 4585). Sections of 14µm were fixed for 10 min using 4% PFA at RT, washed several times with PBS 1×, and blocked using gelatin buffer for 1 h. Sections were incubated with primary antibodies diluted in gelatin buffer overnight at 4 °C. The next day, sections were washed with 0.3% Triton X-100 and incubated with a secondary antibody (1:500) diluted in gelatin buffer for 1 h. Later, these sections were washed with 0.3% Triton X-100, incubated with DAPI for 10 min, and washed with PBS 1×. Sections were mounted using Invitrogen ProLong Diamond Antifade Mounting media. Primary antibodies were used as follows: P-cadherin (Cadherin-3, R&D, AF761; 1:400), Keratin 10 (BioLegend, 905403; 1:1,000), Loricrin (BioLegend, 905104; 1:500), Filaggrin (BioLegend, 905804; 1:400), EDAR (Novus Biologicals, AF745; 1:200), and SOX2 (Cell Signaling Technology, C70B1; 1:200).

Images of whole-mounts and cryosections were captured using an LSM-900 confocal microscope (Zeiss) with either a Plan-Apochromat 20× air objective (NA: 0.8) or a LD C-Apochromat 40× water immersion objective (NA: 1.1). Basic image adjustments were performed in Fiji (ImageJ).

### Quantification of cell proliferation

For cell proliferation assay, pregnant female mice were injected intraperitoneally with EdU (Sigma, 900584), allowing E18.5 embryos to be pulsed for 30 min. Embryos were dissected, and the back skin was embedded in OCT as described above. EdU detection on cryosections was done according to the manufacturer’s instructions (Click-iT EdU Alexa Fluor 647 Imaging kit, Life Technologies, C10340). The ratio of EdU^+^ cells to all cells (DAPI^+^) was calculated for basal (based on P-cadherin^+^) and hair follicle (based on morphology) cells.

### Quantification of skin phenotype

*To* *measure skin thickness,* cryosections of E18.5 back skin were stained with P-cadherin to highlight the basal layer, Keratin 10 and DAPI. Skin thickness was measured using the Fiji straight line tool, as illustrated in Fig 2.

*To assess hair follicle orientation*, cryosections of E18.5 back skin were stained with P-cadherin. The angle between the basal layer and hair follicle was measured using the Fiji angle tool. Hair follicles with an angle greater than 80° were classified as straight. Frequency of hair follicle angle was plotted in a circular histogram using R Studio (2024.04.2) and the R package *ggplot* with bins of 10°.

*To measure hair follicle length*, cryosections of E18.5 back skin were analyzed by drawing a line (straight or segmented) from the bottom of the basal layer until the end of the hair peg, with measurements taken using Fiji.

*To determine the number of hair follicles*, whole-mount preparations of back skin were stained with P-cadherin, which highlights hair placodes, germs, and pegs and their number per square millimeter was evaluated.

*To assess whether PCP is established in the epidermis and placode*, whole-mount immunofluorescence for CELSR1 and E-cadherin was performed using E15.5 back skin. 2D segmentation and polarity analysis for regions of the IFE and placode were performed using the Tissue Analyzer Fiji plugin [56]. The software calculates the angles of these domains and their intensity of polarization. As described in *Leyboya and colleagues*, a custom python code was used to visually represent the data with the length of the red line within each cell indicating the magnitude of intensity and the angle representing the axis of polarity along the anterior-posterior axis of the embryo [4]. Frequency of polarization along the anterior-posterior axis was plotted in a circular histogram using R Studio (2024.04.2) and the R package *ggplot* with bins of 15°.

*To quantify the number of SOX2*^*+*^*, SOX9*^*+*^*, and LHX2*^*+*^ *cells, as well as placode area based on EDAR*^*+*^ *domain and the combined LHX2*^*+*^ *and SOX9*^*+*^ *domains*, whole-mount immunofluorescences for P-cadherin, SOX9, LHX2, SOX2, and EDAR were performed on E15.5 back skin. The area of each placode was measured in Fiji using the freehand selection tool to outline the EDAR^+^ domain. The area of LHX2^+^ and SOX9^+^ domains was measured in the same way, using SOX9 staining to define the outline. Only basal layer cells were included in the analysis, as determined from orthogonal views. The multi-point tool in Fiji was used to count the number of SOX2^+^, SOX9^+^, and LHX2^+^ cells.

*To assess the angle of hair germs,* Z-stacked wholemount images were taken at E16.5 stained with P-cadherin. The orthogonal views were used to identify if the hair follicle invagination was at an angle or straight.

*To measure the mean intensity of E-cadherin, P-cadherin and F-actin at cell-cell junction,* a single Z-stack from the bottom of the basal layer representing placode initiation or IFE region was isolated. 2D segmentation was performed using the Tissue Analyzer Fiji plugin. A custom MATLAB code was used to measure the intensity of P-cadherin, E-cadherin, and F-actin in the segmented region relative to a given position; either center of placode or center of IFE region analyzed [4]. The data was represented using a scatter dot plot.

### Barrier assay

Briefly, E16.5, E17.5, and E18.5 embryos were isolated. Euthanized embryos were immersed in ice-cold PBS 1× for 30 min. Embryos were immersed in a cold methanol gradient (1%–25%, 2%–50%, 3%–75%, 4%–100%) in water, and rehydrated in a methanol gradient in water (1%–75%, 2%–50%, 3%–25%, 4%–0%), taking 2 min per step. Embryos were next immersed in 0.1% toluidine blue solution in water on ice for 2 min, with inversions and destained in PBS 1× to reveal the dye pattern and barrier properties.

### Statistical analysis

Statistical analyses were all performed with Prism 8 (GraphPad Software) unless stated otherwise. In all analysis, experiments were made independently for each pair of embryos (*Arhgef3*^+*/*+^ and *Arhgef3*^*−/−*^; sex as a biological variable was not considered given the embryonic nature of the analysis).

*For proliferation rate based on EdU*^*+*^ *cells for both basal and hair follicle cell analysis*, the total number of EdU^+^ P-cadherin^+^ cells per embryo was normalized with the total number of P-cadherin^+^ cells (based on DAPI staining). The difference between proliferation rates was assessed with a two-tailed Mann–Whitney test with *α* = 0.05 since there were only *n* = 5 individuals per sample.

*For skin thickness and peg length*, measurements were made multiple times on *n* = 5 individuals per genotype and the difference between these two groups was assessed with a two-tailed nested *t* test with *α* = 0.05. Nested *t* test allows to account for individual variability, as multiple measurements are made in each individual.

*For the average number of hair placodes, hair germs, and hair pegs* per 1 mm^2^ region of back skin, a two-way ANOVA was performed followed by multiple comparisons for each hair follicle stage with *n* = 5 embryos per genotype. Sidak’s correction was applied to adjust for those multiple comparisons and adjusted *P* values are reported (with starting *α* = 0.05).

*For hair follicle angle as well as for CELSR1 polarization angle,* the circular mean was calculated using R Studio (2024.04.2) and the R package *circular*. For both datasets, a Watson *U*^2^ test with *α* = 0.05 was performed with R Studio using the package *circular* to compare the distribution of the angles between *Arhgef3*^+*/*+^ and *Arhgef3*^*−/−*^ animals. This non-parametric test (for which the null hypothesis is that the two samples of angles come from the same population) was chosen to account for the nature of the measurements, which are not fully circular in distribution. It is also known to be more robust and powerful than other comparative tests for both angular and directional data. The Watson *U*^2^ test for homogeneity provides a *P* value in confidence interval, and we approximated a *P* value by performing 10,000 permutations of our datasets. This test was performed with *n* = 5 embryos per genotype (hair follicle angle at E18.5), *n* = 3 embryos per genotype (hair follicle angle at P1) and *n* = 3 embryos per genotype (CELSR1 domains angle). We then performed a two-sided Fisher’s exact test with *α* = 0.05 on the same datasets to assess the difference in the number of straight hair follicle (]80.0°,90.0]) and the number of polarized cells (]−15.0°,15.0]), respectively.

*For frequency of straight germ,* the difference between the genotypes was assessed with a two-sided Fisher’s exact test with *α* = 0.05 from *n* = 5 embryos per genotype.

*For the area occupied by the EDAR*^*+*^ *cells and the number of SOX2*^*+*^ *cells below the EDAR*^*+*^ *placodes,* the difference between the genotypes was assessed with a two-tailed Mann–Whitney test with *α* = 0.05 from *n* = 4 embryos per genotype.

*For the number of LHX2- or SOX9-positive cells per placode as well as for their area,* the difference between the genotypes was assessed with a two-tailed Mann-Whitney test with *α* = 0.05 from *n* = 3 embryos per genotype for LHX2^+^ cell count and *n* = 5 embryos per genotype for SOX9^+^ cell count and area.

*For intensity of P-cadherin, E-cadherin, and F-actin in the cells of the placodes*, inner cells were determined to be between 0 and 10 µm from the center of the placode, whereas outer cells were determined to be between 30 and 40 µm from that center. A D’Agostino–Pearson normality test was first performed to assess if the data followed the Gaussian distribution. As none of those datasets did follow the Gaussian ideal, a Kruskal–Wallis test followed by Dunn’s multiple comparisons (of specific pairs) was performed with *n* = 5 placodes (for P-cadherin) or *n* = 6 placodes (for E-cadherin) per genotype and adjusted *P* values are reported (with starting *α* = 0.05). For F-actin intensity, as the staining did not allow for proper segmentation of the whole placode and no difference in the trendlines was observed between *Arhgef3*^+*/*+^ and *Arhgef3*^*−/−*^, a two-tailed Mann–Whitney test (with *α* = 0.05) was performed with *n* = 5 placodes per genotype.

*For intensity of P-cadherin, E-cadherin and F-actin in the IFE,* fluorescence at the junctions was analyzed using linear mixed-effects models to account for repeated measurements within regions and experiments. Data were transformed (square-root) when necessary to meet normality and variance assumptions, and model residuals guided the final model choice. Genotype was treated as a fixed effect, with Region and Experiment as random effects. Pairwise contrasts were computed with the *emmeans* package in R, with Satterthwaite’s approximation for degrees of freedom. The threshold for statistical significance was set at *α* = 0.05 with *n* = 5 IFE regions per genotype for each protein of interest.

## Supporting information

S1 Fig *Arhgef3* transcript variant 3 is the main isoform expressed in the skin. Expression of *Arhgef3* transcript variants in the placode and the epidermis using the *Sulic and colleagues* dataset, shown as mean FPKMs ± SEM for *n* = 7 samples per placode and IFE. Underlying dataset for this figure is available at: https://doi.org/10.5281/zenodo.17400745.
(TIF)

S2 Fig ARHGEF3 controls junctional P-cadherin levels in the IFE. **(A)** P-cadherin whole-mount immunofluorescence of E15.5 IFE. The right panel shows P-cadherin intensity in pseudocolor with the corresponding scale to the right side. **(B)** Graphs display normalized P-cadherin intensity at the cell junction from the center of IFE frame in *Arhgef3*^+*/*+^ (top panel) and *Arhgef3*^*−/−*^ (bottom panel) embryos. Each point represents a cell; black lines point the trend for *n* = 5 regions from 2 embryos per genotype and the experiment was independently performed 2 times (total cells analyzed: *Arhgef3*^+*/*+^ = 278, *Arhgef3*^*−/−*^ = 328). **(C)** E-cadherin whole-mount immunofluorescence of E15.5 IFE. The right panel shows E-cadherin intensity in pseudocolor with the corresponding scale to the right side. Scale bars: 20 µm. **(D)** Graphs display normalized E-cadherin intensity at the cell junction from the center of IFE frame in *Arhgef3*^+*/*+^ (top panel) and *Arhgef3*^*−/−*^ (bottom panel) embryo. Each point represents a cell; black lines point the trend for *n* = 5 regions from 2 embryos per genotype and the experiment was independently performed 2 times (total cells analyzed: *Arhgef3*^+*/*+^ = 308, *Arhgef3*^*−/−*^ = 369). **(E)** Graph displays P-cadherin intensity (a.u.) at cell junction in the IFE as mean±SEM. Statistical analyses were performed using a linear mixed-effects model, *P* value = 0.0103, *n* = 5 regions from 2 embryos (represented by circle and square symbols) per genotype and the experiment was independently performed 2 times (cells analyzed per region: *Arhgef3*^+*/*+^ = 45, 49, 51, 60 and 73; *Arhgef3*^*−/−*^ = 58, 60, 61, 71 and 78). **(F)** Graph displays E-cadherin intensity (a.u.) at cell junction in the IFE as mean ± SEM. Statistical analyses were performed using a linear mixed-effects model, *P* value = 0.4769, *n* = 5 regions from 2 embryos (represented by circle and square symbols) per genotype and the experiment was independently performed 2 times (cells analyzed per region: *Arhgef3*^+*/*+^ = 41, 46, 59, 64 and 75; *Arhgef3*^*−/−*^ = 57, 64, 79, 81 and 88). Underlying dataset for this figure is available at: https://doi.org/10.5281/zenodo.17400745.
(TIF)

S3 Fig ARHGEF3 modulates F-actin organization at cell-cell junctions in the IFE. **(A)** F-actin whole-mount immunofluorescence of E15.5 IFE. The right panel shows F-actin intensity in pseudocolor with the corresponding scale to the right side. Scale bar: 20 µm. **(B)** Graphs display normalized F-actin intensity at the cell junction from the center of IFE frame in *Arhgef3*^+*/*+^ (top panel) and *Arhgef3*^*−/−*^ (bottom panel) embryos. Each point represents a cell; black lines point the trend for *n* = 5 regions from 2 embryos per genotype and the experiment was independently performed 2 times (total cells analyzed: *Arhgef3*^+*/*+^ = 349, *Arhgef3*^*−/−*^ = 253). **(C)** Graph displays F-actin intensity (a.u.) at cell junction in the IFE as mean ± SEM. Statistical analyses were performed using a linear mixed-effects model, *P* value = 0.0010, *n* = 5 regions from 2 embryos (represented by circle and square symbols) per genotype and the experiment was independently performed 2 times (cells analyzed per region: *Arhgef3*^+*/*+^ = 52, 61, 68, 77 and 91; *Arhgef3*^*−/−*^ = 36, 47, 52, 55, and 63). Underlying dataset for this figure is available at: https://doi.org/10.5281/zenodo.17400745.
(TIF)

## Abbreviations

BSA: bovine serum albumin
EdU: 5-ethynyl-2′-deoxyuridine
FPKMs: fragments per kilobase per million
IFE: interfollicular epidermis
ISH: in situ hybridization
PBS: phosphate-buffered saline
PCP: planar cell polarity
PFA: paraformaldehyde
RT: room temperature
